# Mapping of health system functions to strengthen priority programs. The case of maternal health in Mexico

**DOI:** 10.1186/1471-2458-11-164

**Published:** 2011-03-15

**Authors:** Miguel A González-Block, Mariel Rouvier, Victor Becerril, Paola Sesia

**Affiliations:** 1 National Institute of Public Health, Av. Universidad 655 Col. Santa María Ahuacatitlán. Cuernavaca, Morelos, 62100, México; 2 Centro de Investigaciones y Estudios Superiores en Antropología Social. Oaxaca, Mexico

## Abstract

**Background:**

Health system strengthening is critical to ensure the integration and scaling-up of priority health promotion, disease prevention and control programs. Normative guidelines are available to address health system function imbalances while strategic and analytical frameworks address critical functions in complex systems. Tacit knowledge-based health system constructs can help identify actors' perspectives, contributing to improve strengthening strategies. Using maternal health as an example, this paper maps and analyses the health system functions that critical actors charged with formulating and delivering priority health programs consider important for their success.

**Methods:**

Using concept mapping qualitative and statistical methods, health system functions were mapped for different categories of actors in high maternal mortality states of Mexico and at the federal level. Functions within and across maps were analyzed for degree of classification, importance, feasibility and coding.

**Results:**

Hospital infrastructure and human resource training are the most prominent functions in the maternal health system, associated to federal efforts to support emergency obstetric care. Health policy is a highly diffuse function while program development, intercultural and community participation and social networks are clearly stated although less focused and with lower perceived importance. The importance of functions is less correlated between federal and state decision makers, between federal decision makers and reproductive health/local health area program officers and between state decision makers and system-wide support officers. Two sets of oppositions can be observed in coding across functions: health sector vs. social context; and given structures vs. manageable processes.

**Conclusions:**

Concept mapping enabled the identification of critical functions constituting adaptive maternal health systems, including aspects of actor perspectives that are seldom included in normative and analytical frameworks. Important areas of divergence across actors' perceptions were identified to target capacity strengthening efforts towards better integrated, performing health systems.

## Background

The surge of financing to scale-up disease control in the context of weak and failing health systems has led to identify health systems strengthening as the top global health priority [1]. This has spurred new thinking on definitions and approaches to health systems analysis and strengthening, seeking to transcend what is perceived as an over-simplistic categorization of horizontal vs. vertical approaches to health promotion, disease prevention and control. Diverse functions have been proposed to target health system strengthening, most recently governance, financing, health service provision, human resources, technology and pharmaceuticals and information [2], with people at the centre of all components as key players and beneficiaries [3]. However, little attention has been given to understand and act upon the perception that people, service providers and health authorities have of the health system in which they work or whose benefits they expect [4].

Evidence-supported policy making is critical to strengthen health systems in a context characterized by rising health needs, ever more complex health systems, new national and global actors and rapid innovation [5]. Health systems analysis and strengthening should therefore pay attention to increasing the capacity of professionals to make decisions at strategic levels in program and disease control management to enable effective integration to the health system. Research-based evidence can be more easily assimilated when it is translated to meet the needs of professionals working in specific health system contexts, and when it is related to the tacit knowledge that enables them to make decisions on a daily basis.

A number of approaches have been proposed to develop a unified health systems framework, relying on diverse analytical, functional or managerial methods [6-10], although their capacity to support disease control capacity strengthening is still to be demonstrated [11]. Systems-thinking frameworks aim to understand complexity and uncertainty in disease control efforts, to anticipate counter-intuitive relationships across functions and to adapt and scale-up complex interventions with system-wide effects such as directly observed therapy-short course (DOTS) [12,13,3]. Systems-thinking seeks to understand how research evidence can be integrated with tacit knowledge through developing frameworks in close relationship to people and health providers in the field [14]. Eliciting actor and context-specific tacit health system knowledge can be useful for evidence-supported policy implementation where both local and global knowledge have to be integrated.

This paper aims to analyze the characteristics of tacit knowledge on which health personnel in tactical and strategic positions rely to address health objectives. Specifically, the paper seeks to analyze how health system functions are constructed by specific actors and in varying contexts as they face critical decisions to meet problems in disease control through their shared, contextually nuanced tacit knowledge. The paper focuses on maternal health in the most impoverished states of Mexico, which are served by multiple federal public providers and where private care is also available although is mostly inaccessible. State level authorities are responsible since 1995 for the organization of health services for the poor and have mounted together with federal authorities concerted efforts to reduce maternal mortality, in accordance with the MDG 5 commitments. One of the biggest challenges is co-ordination across federal and state authorities and health providers as well as with local authorities and civil society [15,16].

Concept mapping [17] was used to identify health system functions, their degree of classification, the ranking of importance in addressing specific health problems, the ranking of feasibility of improving functional performance and, finally, the structural relationships across functions that code their categorization through underlying contrasts. This last dimension addresses how tacit knowledge builds cognitive maps of functional responsibilities through the systematic opposition and conjunction of categories that help to intuitively identify problems and mount responses.

Research was carried out as part of a research utilization capacity strengthening project for state-level decision makers and program managers in Mexico wishing to focus on disease control for the poor and indigenous populations within their states. Revealing tacit knowledge was a first step in evidence use through identifying their own but varying and often unexamined perceptions of a problem. Maternal health was identified as a common and pressing concern, which could enable them to learn from the project and to extent lessons to other priority programs at a later date.

## Methods

Four states in Mexico were purposively selected for the study, having large rural and indigenous populations and with a patent need for health systems strengthening to achieve specific health-related Millennium Development Goals. The states of Mexico, Guerrero, Oaxaca and Veracruz have a combined population of 27.7 million or 26.9% of the national total, of which 13.6% is indigenous population, a figure that for Oaxaca rises to 22.5%. They are ranked at the bottom of 32 Mexican states in the Human Development Index, save for the state of Mexico, which ranks 19. State Ministry of Health decision makers and experts selected maternal mortality as a common concern for capacity strengthening through the use of evidence. Indeed, the four states account for 28% of the 1218 cases reported for the country in 2009 and with maternal mortality rates per 100,000 live births for the same year ranging between 106.0 in Guerrero and 45.6 in the state of México, as against 56 nationally. The states of Mexico and Veracruz also account for the largest number of maternal mortality cases of any state.

A community of practice was identified within each state, defined as a group of individuals engaged in roles and relationships to create maternal health system knowledge, define a field of expression and research and identify tools and objects for manipulation [18]. The community of practice in each state was limited for practical purposes to around twenty-five participants, while national decision makers and researchers were also included. In aggregate, the sample consisted of a balanced mix of 11 state and 11 federal decision makers (ministers of health, medical and finance/administration directors, delegates for other federal medical programs); 19 system-wide support staff in charge of financing, human resources, planning, quality of care and teaching, research and training; 19 reproductive health program managers and local area health managers in the poorest municipalities with high incidence of maternal mortality; 22 hospital staff in charge of maternal health, and 12 researchers from academic institutions.

A series of three workshops was held in each state between March and April 2010 combining capacity strengthening for evidence utilization and concept mapping. Federal decision makers and researchers participated individually at a later stage. The first set of workshops in the series was attended by 21 participants per state, on average (86 total, with a minimum of 8 in Oaxaca). Within each workshop, a variable number of small groups of participants with similar organizational profiles brainstormed to the question "Which are the health system problems that block access to interventions and tools of proven effectiveness to promote maternal health, prevent disease during pregnancy and avoid maternal mortality?" Ideas were first produce in writing and then read aloud by a moderator to reduce duplicates through discussion.

The 460 ideas produced by small groups were later integrated into 99 unique problems through content analysis and generalization, bringing them to a number that could allow reliable ranking and sorting in the second workshop [17]. Problems were individually printed in 2 × 3 cards with five-point rating scales to measure importance for maternal health ("not important" to "of vital importance") and feasibility of being solved ("impossible"; "difficult"; "possible but no solution known"; "a solution is being formulated"; "a solution is being implemented"). Stacks of cards were distributed to 79 of the original 86 participants and to 15 federal decision makers and researchers. After explaining the purpose of the exercise, each participant proceeded to rank each problem and to sort them into as many piles as they thought important to classify problems for their strategic analysis. The only rules were to produce more than one pile and to allocate each card to only one pile. Participants were asked to name each pile with a descriptive title and to place piles in a named envelope.

Pile data and sorter's role and gender were input to the software Concept Systems Core^@ ^V.4 [19]. Problems were mapped through multi-dimensional scaling and factor analysis for groups with at least 11 sorters to ensure reliable maps [17]. Specific problems were mapped in diametrically opposed positions when they were never or seldom grouped together in the card piles across sorters; problems were mapped more tightly together when they were often piled together, and were mapped in the middle when they were as often as not sorted together. Factor analysis grouped the problems into as many function regions as desired at different levels of aggregation.

Data for all participants was first used to generate national concept maps. Actor data was then processed separately to generate and rank six specific maps, one for each actor at the national level.

After analyzing between 8 and 15 levels of aggregation for the national map, a ten-function map was selected as the most significant and parsimonious. Specific maps across actors were generated at the same level for comparative purposes. The Concept Mapping software assigned the most encompassing label to each function, drawing from the individual pile labels and choosing the one with best statistical fit. Function labels for state-level maps were in some cases modified by state communities of practice at a third workshop, seeking the fullest agreement they thought possible with as many of the specific problems contained within each function. Labels for the actor-specific maps were likewise reviewed and modified by the project team.

Maps and functions within them were analyzed for degree of classification, importance, feasibility and structural position. Degree of classification is the number of problems contained within each function. The larger the number of problems, the more richly classified is the function. The Concept Mapping software ranked each of the ten health system functions by importance and feasibility using the problem rankings of individual participants. The software did this by dividing the average grade differences across functions by five, and assigning a value of one to the lowest rank and a value of five to the highest. Spearman correlations were then run by the software across chosen actors for all ten functions.

Structural relationships between functions were compared within and across maps. To this end, the most commonly identified function across all maps was identified and used as reference to align all maps with this function at the top-centre position. Quadrants were then identified and the functions within each quadrant were grouped and placed as bars in a column, with functions at the centre of maps placed at the bottom. By placing columns besides each other, concept maps could then be compared.

The research protocol was reviewed by the Ethics Committee of the National Institute of Public Health. No informed consent was required. All data was maintained confidential and names of subjects were deleted after their profiles were entered in the database.

## Results

### National concept map

**Figure 1:**
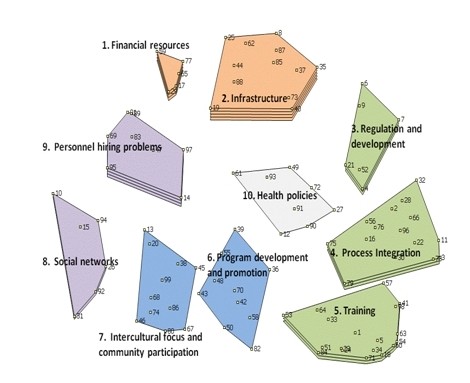
Concept Map of Health System Functions Relevant for Maternal Health, Ranked for Importance. All Actors*. * The number of layers in each function refers to the importance assigned 1 = low; 5 = high. Numbered points refer to the 99 individual health system problems brainstormed by participants.

Functions F4 Process Integration and F5 Training are those with greatest classification (Figure 1, Table 1).

**Table 1:** Average Ratings of Importance (I) and Feasibility (F) of Maternal Health System Problems within Each Function in the National Map, by Actor Group*

				State-Level Actors															
												
Function	No of problems in function	All		Decision makers		System-wide support staff		Hospital staff		Reproductive health		Male		Female		Federal decision-makers		Research ers	
		**I**	**F**	**I**	**F**	**I**	**F**	**I**	**F**	**I**	**F**	**I**	**F**	**I**	**F**	**I**	**F**	**I**	**F**

1. Financial resources	5	4	3	5	2	5	3	3	2	5	2	3	3	4	3	2	5	1	2
2. Infrastructure	12	5	3	5	4	5	4	5	2	5	3	5	3	5	3	4	4	4	4
3. Regulation and development	6	3	2	1	2	4	3	3	1	4	3	2	2	4	2	1	3	2	2
4. Process Integration	15	3	5	4	5	5	4	3	5	4	5	3	5	3	5	3	5	2	5
5. Training	18	3	5	3	5	5	5	2	5	2	5	2	5	3	5	3	5	3	5
6. Program development and promotion	10	1	4	2	3	1	4	1	4	1	5	1	4	1	4	3	4	1	3
7. Intercultural focus and community participation	11	1	4	2	4	2	3	1	3	2	5	1	3	1	4	1	4	1	4
8. Social networks	6	2	1	4	1	4	1	1	1	4	1	3	1	2	1	1	1	3	1
9. Personnel hiring problems	8	4	1	5	1	5	1	3	1	4	2	5	1	4	2	5	1	5	1
10. Health policies	8	1	4	1	5	4	3	1	4	1	5	1	4	1	4	1	3	1	5

* Rank: 1 = low; 5 = high

F2 Infrastructure receives top importance, followed by F1 and F9 Personnel Hiring Problems. F1 and F2 are opposed to functions F5 Training, F6 Program Development and Promotion, F7 Intercultural Focus and Community Participation and F8 Social Networks. The functions opposed to the highly ranked financing and infrastructure all focus on human and social capital and are ranked with the lowest importance, save for F5, which is intermediate. F8 and F9 focusing on processes relating systems to their social context are opposed to F3 Regulation and Development and F4 Process Integration, focusing on internal administrative processes. F10 Health Policies at centre is assigned low importance.

**Figure 2:**
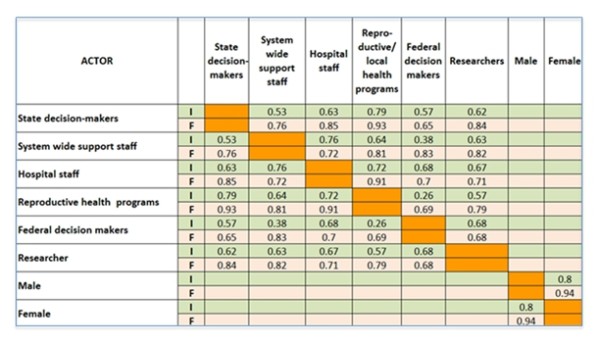
**Correlation Matrix for Importance (I) and Feasibility (F) of Maternal Health System Functions across Actors**.

There is a wide spread in the rank correlations of importance assigned by participants to each function, when compared across actor categories (Figure 2). No such spread is apparent with respect to feasibility. The correlation of importance across actors is highest when comparing male with female participants (r = 0.80). There is a negative relationship -although weak- between the rankings of importance and feasibility across functions (r = -0.14). The correlation of the perception of function importance by federal decision makers is highest -although still moderate- when compared to hospital staff and researchers (r = 0.68 in both cases) and lowest against reproductive health programs (r = 0.26). This latter is of interest given that federal decision makers seek a close relationship with reproductive health and local area program officers on the ground. The correlations of state decision makers against their federal counterparts and against hospital staff are moderate (r = 0.57 and 0.62, respectively), while against reproductive health program managers is moderately high (r = 0.79). Interestingly, it is lowest with system-wide support staff (r = 0.53), suggesting that the mind-set of state decision makers is closer to that of clinicians and public health workers than to administrators. It is also interesting to note that reproductive health program officers have a closer agreement with state decision makers than with federal decision makers. Hospital staff and reproductive health program managers correlate highly (r = 0.72), as do personnel across gender (r = 0.80).

### State decision makers

**Figure 3:**
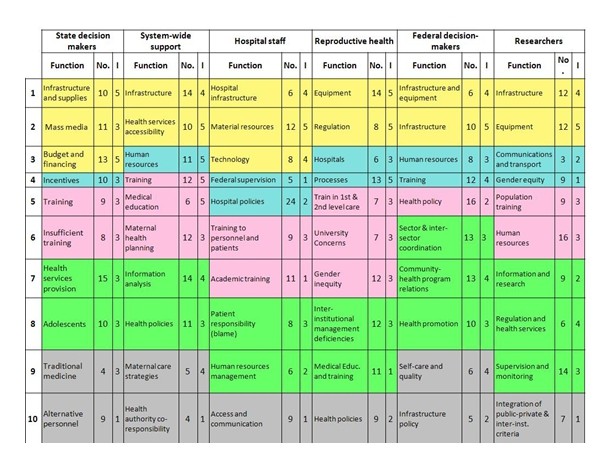
Health System Functions Specific to Each State Actor Category, with Number of Problems (No.) and Importance (I)*. * Colours indicate the position of each function in the actor's map quadrants after placing "Infrastructure" in all maps at the top: Top (yellow); Right (blue;) Bottom (pink); Left (green), and Centre (grey). All maps were rotated to have the infrastructure function at the top. I = Importance is coded 1 = Low; 5 = High.

Analysing now the specific maps for each of the actor categories (Figure 3), functions F1 Infrastructure and Supplies and F3 Budget and Financial Resources have the highest importance and also high classification. F7 Health Services Provision has the highest classification although it is assigned a moderate importance. F1, F2 Communications on Mass Media and F3 -of a material and mass-marketing nature- are opposed to F5 Training and F6 Insufficient Training, focusing clearly and emphatically on human resource processes. F4 Incentives is opposed to F7 and to F8 Adolescents, without a clear meaning. At the centre of the map are placed F9 Traditional Medicine and F10 Alternative Personnel, this last with lowest importance.

### System-wide support staff

Most functions are ranked 4 or 5 in importance. F4 Infrastructure and F5 Health Services Accessibility are opposed to F4 Training and F5 Medical Education, suggesting an opposition between material and human resources. F3 Human Resources is contrasted with F7 Information Analysis and F8 Health Policies and to an extent with neighbouring F6 Maternal Health Planning, all of which focus on health policy and planning and which receive among the lowest importance except for F10 Health Authority Co-Responsibility in the middle, with the lowest importance.

### Hospital staff

Function F2 Material Resources is the most highly ranked for importance. Neighbouring functions F1 Hospital Infrastructure and F3 Technology follow in importance. This set of functions -all of a clear material nature- are opposed to F6 Training to Personnel and Patients and F7 Academic Training, both of a human resource nature and receiving low to very low importance, respectively. F4 Federal Supervision and F5 Hospital Policies receive also very low importance, and are opposed to F8 Patient Responsibility (Blame) and F9 Human Resource Management, which receive moderate to low importance, respectively. However, F5 is the most highly classified function, with a total of 24 problems and the highest for any function across maps. F10 Access and Communication receives also least importance and is placed in an ambiguous position.

### Reproductive health program managers

Function F1 Equipment is ranked of top importance and is highly classified. Also of top importance are neighbouring F2 Regulation and F4 Processes. All other functions are ranked 3 or below, suggesting these actors have a clear set of priorities. At the centre is F9 Medical Education and Training with the lowest importance and F10 Health Policies, ranked of low importance. F1 and F2, with a mix of infrastructure and regulation, are opposed to F5 and F6 focusing on training or in technical support.

### Federal decision makers

Function F2 Infrastructure is given top importance. F7 Community-Health Program Relations is highly classified and assigned a high importance. F10 Infrastructure Policy is placed in the middle, with only a few points and given lowest importance. Infrastructure functions F1 and F2 are placed opposite F5 Health Policy, the most classified function although with low importance. This opposition suggests a contrast between the technical authority held by federal decision-makers and the more unwieldy infrastructure issues that depend on financial and human resources not under their direct control. Human resource functions F3 Human Resources and F4 are placed opposite F6-F8, suggesting a contrast between the centralized control over human resources and functions that are more dependent on state and community level processes.

### Researchers

The function with highest importance is F2 Equipment, being also highly classified. F1 Infrastructure follows in importance. F6 Human Resources is the most highly classified function. The function in the middle F10 Integration of Public-Private and Inter-Institutional Criteria receives the lowest importance, together with F4 Gender Equity. As with other maps, infrastructure and equipment functions are opposed to F5 Training, while social functions F3 and F4 are opposed to health sector process functions F7 Information and Research, F8 Regulation and Health Services and F9 Supervision and Monitoring.

## Discussion

The moderate to high correlations in the ranking of importance in the national map across hospital and program officers, across state decision-makers and reproductive health program officers and across the gender of participants points to a tightly knit, gender-neutral community of practice with respect to maternal health. The lower agreement between federal and state decision makers, between federal decision makers and reproductive health/local health area program officers and between state decision makers and system-wide support officers are of interest and possible concern. The latter situation may respond to the fact that decision makers at the state level are almost always clinicians with little administrative experience.

Health system maps are configured with a fairly constant set of oppositions. Health sector material and structural features -financing and infrastructure- are consistently placed opposite health sector processes and particularly opposite to training. On the other axis, health sector functions of regulation, processes and training are opposed to functions at the margins of the formal health system such as personnel hiring, social networks and community participation. The opposition between infrastructure and training is present across all maps, except for federal decision-makers. The more generic human resources function is mentioned across maps next to diverse functions, including three mentions at the centre of maps, where ambiguous functions are represented.

The mapping of functions along the structures-processes axis suggests that communities of practice perceive certain functions as unwieldy and dominated by federal and state actors outside of their direct control -particularly financing and infrastructure, as opposed to processes that can be managed, particularly the training of human resources. Comprehensive human resource management is more ambiguously conceived as both a structure and a process closely associated to other functions. Such coding reflects a fragmented and still highly centralized system, where federal decision-makers, trade unions and state politicians wield most power, particularly over human resources and infrastructure.

Functions identified at the centre of all maps, while highly ambiguous for the communities of practice, have a strategic importance as they mediate across all peripheral and otherwise less connected functions. This role is thus apparent for alternative health care models (state decision makers), authority co-responsibility (system support staff), alternative personnel (state decision makers) and self-care and quality (federal policy makers).

The health policy function is mapped at the centre and is ranked with low importance for most actors, with the exception of federal decision makers, for whom health policy is highly focused and highly classified. This finding strongly suggest that maternal health policy is dominated by the federal government and that state authorities have little capacity to reformulate it or to enact state policies. This may be a consequence of a still unfinished and highly ambiguous decentralization process where state ministers are political appointees of state governors, but where most policy and financing is federal, particularly in the poorest states of Mexico [15].

Concept mapping proved to be a useful tool to order and understand the tacit knowledge that diverse health sector actors draw-upon when collaborating for a common yet highly complex goal such as the prevention of maternal mortality. The process enabled participants to identify critical functions and to identify their importance, feasibility and relationship to the health system and to its context. By representing results in a graphic way, stakeholders were able to grasp complex relationships in a more intuitive way and to make them available for analysis, as shown. The ranking of importance and feasibility led to discriminate across functions and to support priority setting for the identification of solutions. The interpretation of concept maps thus promises to enable the assessment of areas of common interest while identifying areas of opportunity and also weaknesses in the relationship across actors.

## Conclusions

This research demonstrated the usefulness of concept mapping to reveal the characteristics of tacit knowledge of health system functions that a wide range of actors consider critical to address maternal health. The most relevant and consistent maternal health system functions in Mexican states with high incidence of maternal mortality are infrastructure and training. They are perceived with high consensus and specificity across actors and states, are highly valued and show a consistent mutual opposition that may account for their reciprocal definition and consolidation in tacit knowledge. These two areas of tacit knowledge correspond with the concerns that federal health decision-makers and maternal health experts have stressed in the last decade as being of uttermost concern in Mexico, especially in the poorest states of the country, such as Guerrero, Oaxaca and Veracruz. The federal Ministry of Health has repeatedly remarked on the importance to improve medical skills in attending obstetric emergencies [20,21]. Efforts to expand hospital infrastructure and train medical personnel in emergency obstetric care at the primary and secondary level are among the most important strategies implemented in these states in the last few years [16]. Mapping of the infrastructure and training functions across actors thus attests to the high level of consensus achieved through policy implementation by federal authorities towards.

Health service provision also turned out to be highly relevant in concept maps, although somewhat less focused and showing adaptation to context and actor positions. The reorganization of health services has also been emphasized by the federal maternal health program on the basis of international evidence, which at least since 2003 proposes regionally based, effective functional health care networks [22,23].

Comprehensive human resource planning and social and cultural issues have lower consensus and classification and, above all, appear dispersed in different positions across maps. This suggests not so much an adaptive process but rather a lack of capacity to address such structural and difficult to influence features of the health system. It is worth emphasizing that social and cultural issues are notwithstanding clearly perceived by actors as intrinsic to the maternal health system, while such features are frequently absent in normative international health system frameworks [2]. Finally, health policy -while relevant- shows on the part of state communities of practice a consistent ambiguity and lack of capacity to be addressed. This is a weakness that could affect other large developing countries where decentralization is critical to face maternal mortality. If it is not addressed, it is likely that investments in infrastructure and training may not be efficient, while efforts to establish health service networks may not be sustainable.

The elicitation of the tacit knowledge that actors have of health systems can help to design and implement health system strengthening and scaling-up strategies that can be more easily adapted to varying situations. Mapping of tacit knowledge can help evaluate how actors´ perceptions change after strengthening efforts, leading to the identification of key functions with greater consensus and more focused relations across system levels.

## Competing interests

The authors declare that they have no competing interests.

## Authors' contributions

MAGB designed the overall project and participated in instrument development, workshops and interpretation. MR, VB and PS developed the research instruments, coordinated workshops and participated in the interpretation of data.

All authors have read and approved this manuscript.

## Pre-publication history

The pre-publication history for this paper can be accessed here:

http://www.biomedcentral.com/1471-2458/11/164/prepub
